# Critical Capability Needs for Reduction of Transmission of SARS-CoV-2 Indoors

**DOI:** 10.3389/fbioe.2021.641599

**Published:** 2021-09-29

**Authors:** Jayne B. Morrow, Aaron I. Packman, Kenneth F. Martinez, Kevin Van Den Wymelenberg, Darla Goeres, Delphine K. Farmer, Jade Mitchell, Lisa Ng, Yair Hazi, Monica Schoch-Spana, Sandra Quinn, William Bahnfleth, Paula Olsiewski

**Affiliations:** ^1^Center for Biofilm Engineering, Montana State University, Bozeman, MT, United States; ^2^Integrated Bioscience and Built Environment Consortium (IBEC), Sanford, FL, United States; ^3^Department of Civil and Environmental Engineering, Northwestern University, Evanston, IL, United States; ^4^HWC Inc., Washington, DC, United States; ^5^Biology and the Built Environment Center, College of Design, Institute for Health in the Built Environment, University of Oregon, Eugene, OR, United States; ^6^Department of Chemistry, Colorado State University, Fort Collins, CO, United States; ^7^Department of Biosystems Engineering, Michigan State University, East Lansing, MI, United States; ^8^Engineering Laboratory, National Institute of Standards and Technology, Gaithersburg, MD, United States; ^9^Johns Hopkins Center for Health Security, John Hopkins University Bloomberg School of Public Health, Baltimore, MD, United States; ^10^Department of Family Science and Center for Health Equity, School of Public Health, University of Maryland, College Park, MD, United States; ^11^Department of Architectural Engineering, The Pennsylvania State University, University Park, PA, United States; ^12^Alfred P. Sloan Foundation, New York, NY, United States

**Keywords:** SARS-CoV-2, COVID-19, indoors, transmission, risk reduction and mitigation measures, biosurveillance, human factors, buildings

## Abstract

Coordination of efforts to assess the challenges and pain points felt by industries from around the globe working to reduce COVID-19 transmission in the indoor environment as well as innovative solutions applied to meet these challenges is mandatory. Indoor infectious viral disease transmission (such as coronavirus, norovirus, influenza) is a complex problem that needs better integration of our current knowledge and intervention strategies. Critical to providing a reduction in transmission is to map the four core technical areas of environmental microbiology, transmission science, building science, and social science. To that end a three-stage science and innovation Summit was held to gather information on current standards, policies and procedures applied to reduce transmission in built spaces, as well as the technical challenges, science needs, and research priorities. The Summit elucidated steps than can be taken to reduce transmission of SARS-CoV-2 indoors and calls for significant investments in research to enhance our knowledge of viral pathogen persistence and transport in the built environment, risk assessment and mitigation strategy such as processes and procedures to reduce the risk of exposure and infection through building systems operations, biosurveillance capacity, communication form leadership, and stakeholder engagement for optimal response. These findings reflect the effective application of existing knowledge and standards, emerging science, and lessons-learned from current efforts to confront SARS-CoV-2.

## Introduction

Although SARS-CoV-2 and the subsequent COVID-19 disease are unique, the foundation of knowledge to assess and mitigate the risk of viral transmission in the built environment is robust. In 2013, the US government published science and technology roadmaps (National Science and Technology Council [NSTC], 2013a, b) providing the foundations for biosurveillance and biological incident response and recovery capacities that are relevant to the COVID-19 pandemic. A National Academies of Sciences Engineering and Medicine [NASEM] (2017) consensus report summarized the state of knowledge, identified gaps, and outlined a multidisciplinary research agenda for achieving indoor environments that promote health and prevent disease (Engineering and National Academies of Sciences and Medicine [NASEM], 2017). Since 2004, the Alfred P. Sloan Foundation has funded some 230 projects totaling nearly $80 million to help hundreds of microbiologists, chemists, engineers, architects and building scientists come together to study the ordinary indoor environments where people live, work and play (oral remarks at CLEAN 2020, 2020). A research agenda for viruses in the built environment published in 2020, just months before COVID-19 took hold, identified four priority areas, including identifying and evaluating interventions for controlling viruses indoors (Prussin et al., 2020). These reports provide a framework to understand knowledge gaps and opportunities to reduce transmission of COVID-19 in the indoor environment.

**FIGURE 1:**
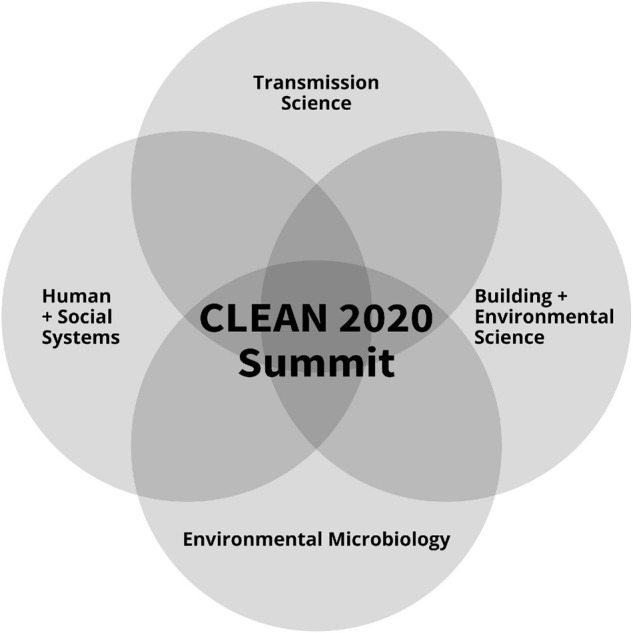
Science Communities Represented in the CLEAN 2020 Summit.

The purpose of this article is to present the findings from a virtual science and innovation summit, CLEAN 2020 (further referred to as “the Summit”), held throughout August, 2020 (CLEAN 2020, 2020). The goal of the Summit was to bring together leaders from business, policy, standards development, science and engineering to (1) understand the current state of the science and knowledge of the factors unique to SARS-CoV-2 transmission and control, and (2) identify opportunities to coordinate science and research to control viral transmission in the built environment. Critical to providing a reduction in transmission is to articulate the research and knowledge gaps to coordinate resources to address dynamic challenges in responding to the COVID-19 pandemic. The Summit is the first of its kind to assess current challenges and pain points felt by industries from around the globe working to safely reopen facilities to customers and employees and inspire innovative solutions to meet these challenges. Research, knowledge and standards development activities across environmental microbiology, building science and engineering, transmission and social sciences (see Figure 1) were discussed, and key findings are reported here.

## Identified Capacity Needs and Opportunities for Coordinated Science and Technology Investment

### Viral Fate, Transport, and Persistence

Elucidation of transmission pathways and associated risks requires knowledge of viral shedding and persistence in the air, on surfaces and in water (Hermesch et al., 2020; Lednicky et al., 2020; Santarpia et al., 2020; van Doremalen et al., 2020). Knowledge of these factors are critical to mitigating viral transmission and enabling effective decision making. Understanding viral distribution and transmission in buildings, even of well-studied pathogens in buildings other than hospitals, has been lacking. This was clear early in the response to COVID-19 when guidance focused on transmission via direct person-to-person contact or indirect surface contact, which underestimated total exposure (particularly via aerosols) and overemphasized the potential for surface decontamination to reduce transmission (How Coronavirus Spreads | CDC, 2020). More information is needed on viral shedding from individuals, infectious dose response, how viruses are transferred from people to building surfaces and air/water systems (notably including bathrooms/toilets/sinks), the persistence of infectious viruses (virions) in these environments, and how people acquire microbes indoors. Experience with related pathogens, such as SARS-CoV-1, suggests that the virus persists in water and infections are transmitted through building air and water systems (Duan et al., 2003; Lee, 2003a, b; Bogler et al., 2020) and that highly localized outbreaks originating from a single resident can spread throughout high-density housing units to entire building complexes and urban neighborhoods (Lee, 2003b; Yu et al., 2014). Recent studies have suggested that persistence of SARS-CoV-2 is similar to SARS-CoV-1 (van Doremalen et al., 2020). SARS-CoV-2 is transmitted through droplets and/or aerosols contributing substantially to superspreader events (Dietz et al., 2020; Fang et al., 2020; Kang et al., 2020; Leclerc et al., 2020; Liu et al., 2020; Morawska et al., 2020; Moriarty et al., 2020; Qian et al., 2020; Rapid Expert Consultation, 2020; Meyerowitz et al., 2021). Most current knowledge of the redistribution of SARS-CoV-2 in the built environment is based on ribonucleic acid (RNA) measurements. Detection and quantification of infective virus is not equivalent to viral RNA. Not all detected RNA is associated with infectious virus, and RNA is generally more persistent than infectious virus in the environment (Bivins et al., 2020; Widders et al., 2020). We need to understand the relationship between RNA detection and infectivity for all types of clinical and environmental matrices (e.g., swab, lavage, sputum, lung sample, feces, air, water, surfaces) as well as how the RNA signal decays in these matrices. Standard methods of testing for the presence of SARS-CoV-2 virions and viral RNA in all relevant media (e.g., saliva droplets, respiratory fluid, colonic fluid, and environmental samples) are still lacking.

The Summit provided a foundation to better understand the potential of airborne transmission of SARS-CoV-2 (Morawska and Milton, 2020) and the evidence supporting source control of other respiratory pathogens (Milton et al., 2013; Leung et al., 2020). The Summit suggested that discrepancies in definitions of aerosols and droplets has caused confusion in both the lay public, engineers and the scientific community. Table 1 contrasts definitions from the infectious disease literature compared with those from aerosol scientists. The Summit discussions yielded proposed definitions for spray-borne transmission to be >100 μm particles or droplets that directly inoculate the eyes, nose, or mouth; and aerosol transmission to include particles or droplets <100 μm that can be inhaled into the lungs.

**TABLE 1:** Current and proposed definitions.

**Current definitions**	**Infectious disease clinicians**	**Aerosol scientists**
Aerosol	Particles less than 5 μm in aerodynamic diameter CDC Environmental Guidelines, 2020.	Solid particles or liquid droplets that are suspended in air. Comprise a wide range of particle diameters (Adhikari et al., 2019).
Droplets	Liquid particles ≥5 μm which primarily fall out of the air quickly (within a few seconds) within 1–2 m from their generation point.	Not defined separately from aerosols. Nonetheless, only aerosol particles or droplets that are visible (>100 μm) deposit within a few seconds and a distance of 1–2 m.
**Proposed definitions:**		
Spray-borne transmission	Particles >100 μm that directly hit the eyes, nose, or mouth	

Performing routine environmental assessments at scale will require validated, standardized, specific, sensitive, and high-throughput methods, coordinated sample-collection workflows, and integration of multiple types of data. Variation in methods is known to result in uncertainty and create broad spreads in analytical results (Downey et al., 2012a, b). The Summit identified gaps in standard methods for (1) sample collection according to validated sampling strategies; (2) sample processing and analysis; (3) interpretation of analytical results; and (4) data integration needed to confidently assess transmission risks and identify the most likely environmental routes of exposure. The Summit reviewed current information on viral fate and transport, surveillance methods, and standards for building infection control. Data were presented on SARS-CoV-2 persistence as a function of temperature and relative humidity (RH) and ultraviolet (UV) light exposure (Biryukov et al., 2020; Ratnesar-Shumate et al., 2020; Schuit et al., 2020). Results to date show inactivation on surfaces is highly dependent on temperature and RH with viral half-life on surfaces at roughly 18 h–1 week depending on surface type under low temperature and low RH, and only a few hours at high temperature and high RH (Biryukov et al., 2020). These data and other recent studies (e.g., Chin et al., 2020; Gormley et al., 2020; van Doremalen et al., 2020; Delikhoon et al., 2021; Harris-Lovett et al., 2021; Marquès and Domingo, 2021) are a start to understanding viral fate indoors; however, additional assessments of viral persistence on a broader range of surface types including porous surfaces and textiles of various composition and age are required. The Summit discussed the likelihood of fomite transfer rates to be consistent with infectious viruses between people and surfaces (Ansari et al., 1988; Mbithi et al., 1992; Thomas et al., 2014; Hirose et al., 2020). These data as well as recent decontamination studies (Kampf et al., 2020) will inform strategies to determine the extent of contamination and guide public health and safety actions such as cleaning, quarantine, isolation, and/or testing. Data integration that includes viral persistence in air, water and wastewater can be applied in real-time modeling tools to support biosurveillance operations to identify new outbreaks, emergence of new or mutated viral pathogens, and guide building and facility operations including air handling, air treatment and surface disinfection.

### Reducing the Risk of Exposure and/or Infection

Methods to reduce the risk of exposure or infection are essential for rapid recovery from a pandemic or other infectious disease transmission incident. Risk-reduction strategies include actions such as quarantine, social distancing, and other measures to mitigate the transmission of disease including cleaning, hygienic practices, decontamination and building air handling operations to mitigate contamination. Quantitative estimates of risk and characterization of the certainty of those risk estimates will inform decisions as to which risk-reduction strategies will be most effective. For example, current guidance on the use and application of existing decontamination methods and strategies is limited in application scenario and may not address or be appropriate for all exposure reduction measures needed (EPA, 2021). The Summit focused conversations on the hierarchy of controls approaches to reducing transmission, development, and integration of decontamination methods and understanding the process for technologies to demonstrate safety and efficacy, aerosol transmission reduction, and building operations.

#### Engineering Controls for Reduction of Aerosol Transmission

Within the hierarchy of controls, the top level of exposure control is elimination of the infectious agent. Specific building design and engineering controls can be applied that contribute to elimination or reduction of exposure to viral particles such as filtration, transmission barriers (e.g., masks, plexiglass partitions), and inactivation and dilution with outside air. Control banding can also be an effective tool for reducing exposure when there are uncertainties in exposure routes. Control banding involves a generic technique that determines a control measure based on a range or “band” of hazards and exposure levels; in essence, a qualitative assessment for determining the level of risk for a specific job and workplace. It applies a bands hierarchy to define the appropriate type or mix of control measures to put in place. Control banding can be appropriately applied to SARS-CoV-2 protection due to the lack of exposure limits or until there is a clear indication of minimal infectious dose (Van Damme et al., 2021). It can also be used to reduce exposure to agent-containing aerosols to increasingly lower levels by selecting additional control strategies such as engineering controls including air filtration and cleaning, and barrier protections, for example, from the source and pathway categories and thus reducing the reliance on personal protective equipment (PPE) (Centers for Disease Control and Prevention [CDC], 2021).

High Efficiency Particulate Air (HEPA) portable air cleaners can help to decrease the airborne concentration of small particles within a room, but they will not protect an individual that is in close proximity to someone who coughs or sneezes. Barrier protections such as plexiglass partitions can help decrease the transmission of large particles that have been disseminated through spray-borne mechanisms such as coughing or sneezing but for smaller particles, the impact becomes limiting and may interfere with designed airflow. The Summit provided information on the efficacy of face coverings as a combination of filtration and barrier controls to reduce the bioburden to the space. Studies have shown that face coverings (cloth and surgical masks) can effectively reduce particle emissions even at the small size range, this effectiveness assumes a good face fit (Asadi et al., 2020; Moghadas et al., 2020). The fabric captures particles exhaled by the wearer, but face seal leakage (e.g., gaps at the cheeks) may allow many of the smaller particles (those inhaled deeply into the lungs) to escape. The corollary to particle escape through gaps is preferential flow during inhalation through gaps. The discussion drew attention to the use of face coverings and a false sense of safety resulting in the wearer overly relying on face coverings and subsequently not applying other basic infectious disease control efforts such as social distancing and avoidance of high-risk environments.

Additionally, installation of disinfecting ultraviolet germicidal irradiation (UVGI) systems (either inside a room or embedded within the ventilation system) can help to reduce the concentration of active aerosolized pathogens (Jensen et al., 2005; Centers for Disease Control and Prevention [CDC], 2009), however, caution must be taken to reduce potential skin/eye exposure when utilizing UV systems. Increasing the supply of filtered outside air into a room by various means can also significantly reduce and dilute airborne particle concentration. Increasing the filtration efficiency of the HVAC system by switching to a higher Minimum Efficiency Reporting Value (MERV) filter will promote the reduction of indoor fine particles. Ventilation and filtration are potentially effective mechanisms to remove exhaled particles from indoor air that avoid the unintended chemical consequences of some of the biocidal treatments described next. It must be noted that first the UVGI and MERV units need validation and careful consideration for application to real-world buildings and usage including impact on power consumption due to the increased energy needs of dense filters.

#### Decontamination and Disinfection

A decontamination or disinfection method is evaluated for its potential to inactivate the pathogen or biocidal efficacy. Standard methods exist to validate the efficacy of biocides used in surface decontamination (ASTM International methods E1053-20, and E2721-16). Products that have met the performance guidelines for effective surface decontamination can be found on the U. S. Environmental Protection Agency’s (EPA) List N (EPA, 2020). List N posting requires testing according to the chemical formulation of the biocide, application procedure and validation of label instructions. Biocidal efficacy is a function of both concentration and time; labels prescribe contact time for a certain kill rate and inadequate contact time may severely hamper the efficacy of the biocide and the kill of the microorganism (or destruction of the virus). Thus, not only must the efficacy be validated, but also the approach used to apply the biocide. The standard methods were originally designed for enveloped viruses—surrogates were used based on the understanding that they predict how the biocides will work against SARS-CoV-2. The Summit discussion considered issues and challenges with surrogate models for SARS-CoV-2 in determining biocide efficacy and differences between validated performance on non-porous surfaces, compared to porous surfaces such as textiles. Additionally, novel and emerging biocides were discussed for which application methods are inconsistent with the method used to qualify the biocide for inclusion in List N. These include the growing use of spray or fumigation techniques, novel air cleaning approaches, such as room fumigation with disinfectants and ozone or hydroxyl radical generators, now on the market. These products are not biocides and are not listed in List N as verification of disinfection chemistry performance is required and standard methods for air cleaning approaches are needed.

The Summit discussion included an assessment of the growing interest in using UVGI to deactivate viruses on either surfaces or airborne particles. Laboratory studies suggest that UV can be effective for hard surface disinfection (Raguse et al., 2016). However, organic materials (e.g., biofilms, phlegm or respiratory fluid, nasal secretions) reduce UV penetration, and thus lowers the efficacy of UV light (Nerandzic et al., 2014; Cadnum et al., 2016). Further, certain types of UV light can be dangerous to human health or potentially cause indoor photochemistry. UV efficacy is also a function of wavelength, distance from the surface, surface type and may be pathogen specific (Mitchell et al., 2019). Initial studies of persistence and sensitivity to disinfection have indicated SARS-CoV-2 behaves similar to other enveloped viruses (Morris et al., 2020; van Doremalen et al., 2020; Marquès and Domingo, 2021). However, studies of the efficacy of UV light against aerosolized microbes and SARS-CoV-2 in particular are limited and more data are required to better understand opportunities for broader application of germicidal UV.

EPA’s List N provides critical information necessary for decision-makers for the appropriate and efficacious selection of biocides for surface decontamination strategies. However, other critical parameters to consider when selecting what biocide to use include impacts on human health, the environment, and materials. Biocides that are often the most effective are often the most toxic or damaging (Mattila et al., 2020a, b). Bleach and some of these other cleaning products can chemically react with material surfaces, potentially causing long-term damage which will result in increased operating costs and environmental consequences (Hora et al., 2020). Balancing the need for immediate and effective decontamination with the long-term unintended consequences from chemical exposure was a focus of discussion for the Summit. Further research into secondary reactions or chemical byproducts from decontamination are warranted, and regulatory agencies should consider these consequences as part of any product assessments and include this information on the product’s label so that the consumer may make an informed decision on its optimal use.

The Summit recognized that initial urgency around surface decontamination was similar to an acute infection but responding to the SARS-CoV-2 pandemic aligns more as a chronic contaminant for which we need to develop long-term approaches to continually manage and contain this virus. The Summit uncovered several research needs focused around long-term cleaning strategies that balance effective decontamination with materials, environmental and health considerations. Specifically, we need:

• Clean-in-place validation: how often do surfaces actually need to be cleaned? Which material surfaces are more susceptible to damage with different disinfectants?
• Methods to validate new technologies to demonstrate their efficacy.
• Ways to test the impact of cleaners on materials, environment and health in real-world environments that consider the proper use of a disinfectant for the appropriate situation and the potential for secondary chemistry and other complex interactions, including potential interaction between disinfectants.

#### Processes and Building Systems Operations

We can reduce viral transmission through a combination of engineering controls including ventilation, filtration, and other supplemental air cleaning applications. Buildings have been designed and operated for thermal comfort and indoor air quality since the energy crisis, for energy efficiency. Ventilation rates in many buildings are too low and filter efficiencies in re-circulated airstreams is too low to properly control infectious aerosol concentrations. Indoor air quality standards for non-healthcare buildings focus on control of building-generated contaminants and human body odor with the goal of achieving acceptable perceived air quality and do not necessarily provide adequate filtration for preventing aerosol transmission and protection against infection. Furthermore, once a building is commissioned, if it was commissioned, there is often too little attention or resources to ensure ventilation systems are operating properly. Going forward, there must be improvements in the design and operation of the built environment to minimize disease transmission, improve air quality, and ensure occupant health as much as reasonable. The Summit discussion referenced the basics of ventilation–delivering outdoor air to occupants and filtering recirculated air and the need for a renewed investment to ensure operational performance. The American Society of Heating, Refrigerating and Air-Conditioning Engineers, ASHRAE, has provided guidance for building system operations including a combination of: increase ventilation and reduce recirculation if feasible (meet code requirements at a minimum), employ higher filtration efficiencies (MERV 13 preferred), maintain design temperature, and RH% and use supplementary UV-C and portable HEPA air cleaners (ASHRAE, 2020; Persily and Ng, 2020).

Airborne transmission of SARS-CoV-2 has been generally accepted by scientists as a significant component of the COVID-19 pandemic (Morawska and Milton, 2020), and recent studies have implicated indoor transmission as a major factor in both local COVID-19 outbreaks and the global spread of the pandemic (Prather et al., 2020; Fang et al., 2020; Leclerc et al., 2020; Qian et al., 2020; Meyerowitz et al., 2021). However, limited information is available on SARS-CoV-2 transmission through building HVAC systems and infectivity via this pathway is unknown (Dietz et al., 2020; Fang et al., 2020; Horve et al., 2020; Meyerowitz et al., 2021). Nevertheless, dilution of indoor air via properly filtered outdoor air remains critical to reducing infectious disease transmission, particularly in settings such as hospitals (Allen and Marr, 2020; Dietz et al., 2020; Morawska et al., 2020). While the number of supply air changes (outdoor air plus any filtered recirculation air) present in many hospitals (typically 6 h-1–12 h-1) cannot be reached in many commercial buildings such as offices and schools, there are controls (e.g., masks, filtration) that can help increase the equivalent air changes in commercial buildings and thus reduce exposure to airborne aerosols (Dietz et al., 2020; Prather et al., 2020). When modifications to HVAC systems are contemplated in order to reduce pathogen transmission risk, it is important to consider a holistic approach. Applying any risk mitigation strategy (e.g., increased outside air, increased filter efficiency, increased air changes) in isolation may have unintended consequences. For example, increasing filter efficiency may reduce total air changes if the fan systems cannot overcome the increased pressure drop. Similarly, increasing outside air to reduce the fraction of recirculated air may result in a lower total airflow and increase energy consumption in order to maintain thermal comfort.

Currently vacant buildings will 1 day be re-occupied and many people are paying more attention to ventilation. Occupants may demand more information about the HVAC systems serving them. While accurate real-time monitoring of particulate matter smaller than 2.5 μm (PM 2.5) and volatile organic compounds (VOC) are largely cost prohibitive now. Similarly, consumer grade PM 2.5 sensors PM 2.5 (Wang et al., 2020) may offer qualitative evidence of system performance. While these sensors do not monitor pathogens directly, integrating particle sensors into a building operational processes can be useful and begin to pave the way for the future of indoor biosurveillance.

### Establishing Biosurveillance Capacity

A clear recommendation from the Summit is the need to develop strategies for comprehensive surveillance of air, water, and surfaces to assess COVID-19 re-emergence as well as new pandemic and epidemic viruses. Surveillance for disease agents, or biosurveillance, is an active data-gathering effort that relates disease activity to threats to human or animal health in order to achieve situational awareness of disease activity and provide an early warning of emerging threats (National Science and Technology Council [NSTC], 2013b). Threat detection must be coordinated with decision-making bodies and communicated with the public in order to prevent, manage or mitigate disease (Planning Committee on Information-Sharing Models and Guidelines for Collaboration: Applications to an Integrated One Health Biosurveillance Strategy—A Workshop, Board on Health Sciences Policy, and Institute of Medicine, 2011). Traditional surveillance systems used by public health for detecting and responding to infectious disease outbreaks often operate with considerable delay (e.g., diagnostic testing delays are well documented), thus complementary biosurveillance of the built environment is needed to reduce the time to information and inform decisions and actions. The Summit identified a need for coordinated surveillance of schools, workplaces, and other high-risk facilities (e.g., hospitals, senior living facilities, and cruise ships). The Summit panel discussions reviewed current efforts to develop surveillance strategies that include monitoring humans, wastewater, building surfaces, and air handling systems. Current data streams for surveillance of SARS-CoV-2 include public health laboratory testing, public health contact tracing, rapid detection on building surfaces and in air systems, as well as building and municipal wastewater systems. However, these data streams are currently analyzed separately, which hinders threat assessment and response. The Summit discussed the needs for integrating these data streams, as well as other critical innovations to expedite information flow, increase accuracy and repeatability, and enhance situational awareness and confidence in reopening. There is a clear need for an integrated population-level biosurveillance program that includes human diagnostic testing, human screening, biosurveillance at multiple scales including indoor spaces (surfaces and air), building scale (air, wastewater and wastewater vent stacks), campus or neighborhood scale (wastewater from multiple buildings), and community/city-scale surveillance (sentinel locations). A strategy for coordinated and sustained surveillance of the built environment and people is needed in order to implement a real-time, data-driven decision-making platform that can quickly respond to disease outbreaks.

Currently, several public and private organizations are conducting building surface surveillance and developing data-driven action plans. During the COVID-19 pandemic, many academic institutions (Betancourt et al., 2020; Barich and Slonczewski, 2021; Gibas et al., 2021; Harris-Lovett et al., 2021) and jurisdictional authorities (Gonzalez et al., 2020; Larsen and Wigginton, 2020; Medema et al., 2020; Peccia et al., 2020) around the globe rapidly developed and deployed wastewater and building air HVAC surveillance tools (Hermesch et al., 2020; Horve et al., 2020). These data sets provide the opportunity to develop best practices and validate sample collection and processing methods. While many surveillance methods have been deployed, integrated data-driven response protocols and risk mitigation strategies are still needed (Larsen and Wigginton, 2020; Thompson et al., 2020; Harris-Lovett et al., 2021). Critical to underpinning decision-making is to know how viral RNA concentration corresponds to concentration of infectious virus, how long viral RNA persists in the built environment and the understanding of how viral RNA persistence is related to infectiousness. Additionally, there are limited common requirements for surveillance programs and decisions made with surveillance data (Aguiar-Oliveira et al., 2020; Centers for Disease Control and Prevention [CDC], 2020) and they are not well coordinated globally. To foster an integrated response, there is a clear need to develop standardized methods, a common data archive and metadata standards for all SARS-CoV-2 environmental results. Working to standardize these approaches will help establish monitoring tools that can be broadly and consistently deployed to facilitate both building/facility-level and community-level responses.

Furthermore, recognizing the role of ecosystem dynamics and the impact of ecosystem health on incidents of spillover events and overall fate and transport of zoonotic diseases is foundational to biosurveillance strategies. The Summit discussion introduced the role of ecosystem health and other enabling conditions pivotal to viral spillover from bat populations (Plowright et al., 2014). Plowright et al. (2017) proposed ecological changes including deforestation that result in stress in bat populations (e.g., scarce food resources) drives the redistribution of bat populations where spillover events can occur. The role of transmission of viruses from animals to humans requires exposure and susceptibility of recipient hosts (e.g., including livestock, wild animals and humans) as well as survival of the virus in the environment post bat shedding (Plowright et al., 2017). A key finding of the Summit discussion on biosurveillance is the need for ecosystem monitoring in order to elucidate the impact of ecosystem health on emergence of novel diseases like SARS-CoV-2.

### Risk Assessment and Mitigation Strategy

Risk and exposure reduction require data and information accumulated through a comprehensive risk assessment process. Quantitative microbial risk assessment (QMRA) is a computational method for integrating the hazard characteristics of infectious agents (e.g., viruses) in macro- and micro-environments with human activities that result in exposure (Quantitative Microbial Risk Assessment, 2020, 2nd Edition | Wiley). QMRA also predicts the risk of infection or illness outcomes associated with these exposures. Within the larger framework of risk analysis, risk assessment methods can be used to effectively design and evaluate risk management strategies based on their likely impact on risk reduction and lead to informed risk communication. Risk assessment is also used to prioritize exposure pathways within an environment and therefore inform decisions about which risk mitigation efforts are most appropriate based on an understanding of the fate and transport mechanisms of pathogens within and on various environmental matrices involved in the transmission pathway (i.e., air, surfaces, food, etc.) Hence, risk assessment requires the integration of multiple data streams and models that describe biological (excretion rates, exhalation/inhalation rates, decay/persistence etc.), physical (aerosolization rates, transport, transfer rates, etc.) and chemical (cleaning, disinfection, etc.) processes leading to environmental concentrations when specific conditions are present at the point of exposure. An exposure dose is then calculated based on the magnitude or extent of contact, duration and frequency of human activities. The likelihood of an adverse health outcome (infection, illness or death) at given exposure levels also follows a quasi-mechanistic approach defined by a dose-response model. Such models account for the distribution of pathogens within the matrix and the probability that a pathogen can survive to initiate infection reaching a target receptor. While models have been developed for highly infectious viruses through the inhalation route previously (Watanabe et al., 2010; Mitchell et al., 2020), a SARS-CoV-2 specific model has not yet been developed. In the absence of a dose-response model, the Wells-Riley equation has been used for simple and quick assessments for airborne pathogens including SARS-CoV-2 (Sze To and Chao, 2010). However, the validity and generalizability of the underlying assumptions of this approach are unknown, and data are needed to establish the relationship between exposure dose and risk of infection for SARS-CoV-2 in order to move forward with less uncertainty in QMRA models. During the Summit, there was some discussion about models and several tools for exposure and risk quantification that allow for quick assessments in specific environments, under certain environmental conditions and social/behavioral components of exposure (population density/duration of time spent in the room) (Kasibhatla et al., 2020; Rocha-Melogno et al., 2020). Several others have emerged in the gray literature or situationally to address the need to assess risks quantitatively (and/or semi-quantitatively), but the full capacity of risk assessment approaches have yet to be brought to bear to address COVID-19 which was identified as a critical gap. Quantitative assessments of risk are needed to determine likely exposure doses of viral particles through each exposure pathway (air and surfaces) and to estimate actual risk reduction provided by a suite of technologies (e.g., disinfection) and operational practices (e.g., air handling, surface cleaning). These assessments would incorporate both inherent variability and uncertainty in order to lead to better decision making and communication as determining an acceptable level of risk is based on assessing both expected values and levels of certainty. Finally, these assessments are needed to provide more comprehensive information for specifying risk mitigation strategies, critical control points for multi-barrier approaches that can be used in concert with epidemiological-based models which focus on testing, isolation, quarantine and eventually vaccination strategies.

### Communication, Leadership, and Stakeholder Engagement

Critical to the success of any risk reduction strategy is insight from the social and behavioral sciences. Throughout the Summit opportunities for science communication and organizational leadership to shape the understanding of the risk and risk perception, decision-making and collective actions to begin to safely resume normal activities were discussed. Engineering controls and management practices to reduce viral transmission in the built environment are more protective, and perceived as more protective, when the system’s design accounts for the concerns, values, behaviors, and information needs of a building’s occupants—whether workers, students, or patrons. Given that knowledge of human factors is essential to safe congregation, “people” experts (i.e., social, behavioral, and communication scientists) as well as epidemiologists, microbiologists, and building engineers/scientists are necessary to develop effective indoor air quality approaches. Summit panelists who addressed the human elements of safe indoor congregation spotlighted three main challenges and outlined some best practices:

• Communication—A growing reliance on social media and the nature of the COVID-19 threat (e.g., difficulty in seeing the cause-and-effect relationship between mitigation measures and health outcomes, binary thinking about public health and economic matters) has produced a complex communication environment. A clear need exists for better coordination and deployment of information resources. Federal strategy and guidance are important drivers for effective local and regional response; in the absence of effective governmental structures, individual companies, schools, and facilities have instituted *ad hoc* measures, and infection control managers have been left to share information via their own networks.
• Stakeholder Engagement—Top-down, one-sided conversations about issues regarding safe congregation have a limited capacity to achieve necessary behavior change or to engender public confidence in protective measures. Instead, it is important that end users of the built environment have an opportunity to provide feedback into mitigation planning and that they feel listened to and respected. Decision makers must be willing to understand users’ values and beliefs. In doing so, they will be in a better position to understand and leverage group norms that shape adoption of personal protective measures like mask-wearing and physical distancing.
• Leadership—The delivery of factual information alone, including that regarding personal protective behaviors and system-level protections, will not create the desired change among building occupants. Emotions are present. Leaders, for instance, should express empathy for users’ health and safety concerns. In addition, leaders and managers should positively model the behaviors that they want to see in their workforce, customer base, or other constituencies.

## Conclusion

The Summit was impactful because it brought disparate groups together that do not commonly work together to meet the changing needs of the community and incorporate lessons-learned into best practices that are holistic. The research community has come together in unprecedented ways to respond to the pandemic. Such focused efforts can be used to guide basic research funding priorities and coordinate funding for identified capability gaps as well as serve as the foundation for future policy development. The research community will continue to benefit from coordinating what is known about buildings and viruses including the unique findings from studying SARS-CoV-2, and rapidly sharing the gaps in our collective knowledge using tools like virtual workshops.

## Author Contributions

All authors listed have made a substantial, direct and intellectual contribution to the work, and approved it for publication.

## Conflict of Interest

KM and YH were affiliated to the commercial company HWC Inc. The remaining authors declare that the research was conducted in the absence of any commercial or financial relationships that could be construed as a potential conflict of interest.

## Publisher’s Note

All claims expressed in this article are solely those of the authors and do not necessarily represent those of their affiliated organizations, or those of the publisher, the editors and the reviewers. Any product that may be evaluated in this article, or claim that may be made by its manufacturer, is not guaranteed or endorsed by the publisher.
